# Green Flame-Retardant Composites Based on PP/TiO_2_/Lignin Obtained by Melt-Mixing Extrusion

**DOI:** 10.3390/polym14071300

**Published:** 2022-03-23

**Authors:** Marlene Andrade-Guel, Christian Cabello-Alvarado, Carlos Alberto Avila-Orta, Marissa Pérez-Alvarez, Gregorio Cadenas-Pliego, Pamela Yahaira Reyes-Rodríguez, Leopoldo Rios-González

**Affiliations:** 1 Centro de Investigación en Química Aplicada, Saltillo 25294, Coahuila, Mexico; marlene.andrade@ciqa.edu.mx (M.A.-G.); carlos.avila@ciqa.edu.mx (C.A.A.-O.); marissa.perez@ciqa.edu.mx (M.P.-A.); pamela.reyes@ciqa.edu.mx (P.Y.R.-R.); 2 CONACYT—Centro de Investigacion y de Innovacion del Estado de Tlaxcala, Tlaxcala 90000, Tlaxcala, Mexico; 3 Departamento de Biotecnología, Facultad de Ciencias Químicas, Universidad Autónoma de Coahuila, Saltillo 25280, Coahuila, Mexico; leopoldo.rios@uadec.edu.mx

**Keywords:** composite, polypropylene, titanium dioxide, lignin, green fire retardant

## Abstract

Nowadays, highly flammable and harmful plastic materials are used in many daily applications. To prevent burning of materials, other harmful molecules or materials that are not environmentally friendly are added to plastics. To overcome this environmental issue, new materials have been investigated. Lignin, an industrial by-product, is an abundant biopolymer that can be used in fire safety plastics; it is considered a renewable and readily available resource. In this work, PP–TiO_2_/lignin composites were obtained with TiO_2_/lignin mixtures through the melt extrusion process, with different weight percentages of nanoparticles (10, 20, 25, and 30 wt.%). The PP–TiO_2_/lignin composites were characterized by XRD, FTIR, TGA, and SEM. Furthermore, cone calorimetry tests and the mechanical properties were evaluated. Cone calorimetry tests revealed that the introduction of 25 wt.% TiO_2_–lignin to the PP matrix reduced the peak of heat release rate (PHRR) and total heat release (THR) by 34.37% and 35.45%, respectively. The flame retardancy index (FRI) values of the composites were greater than 1.0 and were classified as *good*; the highest value of 1.93 was obtained in the PP-30 sample. The tensile tests demonstrated that the flexural modulus of the composites increased gradually with increasing lignin and TiO_2_ content, and the flexural strength decreased slightly. The use of lignin in PP composites can be an excellent alternative to synthesize new materials with improved flame-retardant properties and which is friendly to the environment.

## 1. Introduction

More than 6000 people died in Mexico over the last 10 years due to the fact of exposure to uncontrolled housing fire, exposure to ignition of highly flammable materials, as well as from explosions or from contact with hot liquids [1]. One fire prevention measure is to promote the use of flame-retardant materials that delay and inhibit combustion. These materials are absorbed and eliminate the space for the action of oxygen in order to stop the fire from spreading, allowing the object to be consumed at a slower rate, giving time to extinguish the fire [2].

Currently, there are different flame retardant additives on the market with various chemical compositions. Halogenated additives possess the best characteristics to combat fire. However, they do not comply with new environmental directives, such as REACH (Registration, Evaluation, Authorization, and Restriction of Chemicals, establishing a European Chemicals Agency), WEEE (Waste Electrical and Electronic Equipment Directive), and RoHS (Restriction of Hazardous Substances), which regulates the use of such materials [3]. Flame retardant additives based on non-halogenated substances have also been used. For example, aluminum trihydrate together with other metal hydroxides, such as magnesium and phosphorus-based additives, are used in a large number of products on the market [4]. 

The regulations mentioned above restrict flame-retardant chemical compounds to avoid adverse effects on living organisms and on the environment. Some green flame retardants have been investigated to contribute to environmental care and are sustainable materials [5]. One of them is lignin, which is a by-product of the process to produce bioethanol, and it is obtained from the discarded biomass of lettuce agave collos, which dries and becomes waste. Lignin is a three-dimensional polymer of phenylpropane bound by carbon–carbon bonds or peroxide bonds; its composition (ash-free) is approximately 62% in C, 32% in O, and 6% in H [6].

Some new materials that can be used as flame retardants are polymeric compounds due to the characteristics conferred by the materials that compose them and the homogeneity of their properties throughout their volume. The combination of multiple materials represents an innovative and effective strategy to create high-performance flame-retardant polymer compounds [7]. Composite materials of polypropylene with TiO_2_/carbon nanotubes have shown good results in decreasing its peak HRR from 1529.53 to 1079.94 kW/m^2^ [8]. Natural fillers, such as halloysite nanotubes, also improve the flame-retardant properties of polyurethanes; the use of low-toxicity compounds in polymers is a good alternative for environmental protection [9], another strategy that improves the fire resistance of polyurethane is the use of phosphorous–nitrogen flame retardants and titanium carbide [10]. Lignin is used to improve the flame-retardant properties of synthetic and biodegradable polymers due to the fact of its excellent thermal properties; its decomposition occurs in a temperature range of 200–500 °C, depending on the nature of its production and chemical modifications of its structure [11,12,13,14]. Klapiszewski et al. studied the thermal stability and mechanical properties of the polypropylene (PP)/silica–lignin composites; the results showed that with the incorporation of silica, these properties were improved [15]. Materials based on lignin or TiO_2_ and polypropylene with good flame retardant properties have been reported separately in the literature [8,16,17,18,19]. The aim of this study was to evaluate the flame-retardant behavior of a composite based on PP, lignin, and TiO_2_ produced by melt-mixing extrusion, since this method can produce homogeneous dispersion and is considered ecologically and economically viable for the production of large volumes of production at the industrial level [20,21]. This paper shows the evaluation of composites based on polypropylene, titanium dioxide, and lignin; the latter was obtained as a by-product of biomass to obtain bioethanol (natural residue). Polymeric composites were obtained by melt-mixed extrusion at different concentrations of 10%, 20%, 25%, and 30% by weight (wt.%) for evaluation as flame-retardant materials and mechanical properties.

## 2. Materials and Methods

### 2.1. Materials

Lignin was provided by the Department of Biotechnology of Universidad Autónoma de Coahuila (Saltillo, Coahuila, México), and it was obtained as a by-product of the process to obtain bioethanol, which was collected and dried at 60 °C for 48 h and ground to a size of 2 mm. TiO_2_ nanoparticles were provided by Chemours Co., (Altamira, México) with a particle size of approximately 200 nm. The polymeric matrix (PP) with a fluid index of 0.76 g/10 min was supplied by Polímeros Nacionales (Ciudad de México, México).

### 2.2. Synthesis of PP/TiO_2_/Lignin Composites

**Figure 1:**
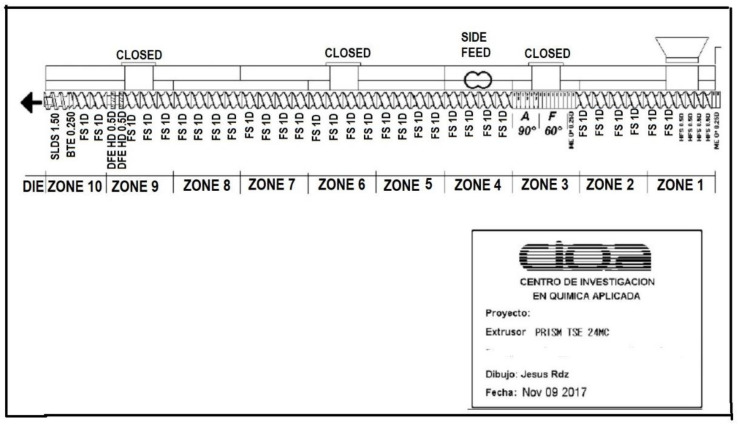
Screw mixing settings used during extrusion.

Melt-mixing extrusion was used to prepare PP composites with a mixture of TiO_2_/lignin (Table 1), using a lab-size twin-screw extruder from Thermo Scientific Model Prism TSE-24MC with a screw diameter of 24 mm, an L/D ratio of 40:1, a temperature profile of 200 °C, and a rotational speed of 100 rpm. Low shear strengths were used to improve the particles’ dispersion in the polymeric matrix in the screw configuration, which is shown in Figure 1. The ratio of the TiO_2_:lignin fillers was 2:1, which in high weight percentages of both fillers was expected to have a significant impact on the flame-retardant properties.

### 2.3. Characterization

Fourier transform infrared (FTIR) analysis was performed with a Magna Nicolet 550 spectrometer (GMI, Minneapolis, Minnesota, USA) using 100 scans and a resolution of 16 cm^−1^ in the range of 4000–400 cm^−1^. The X-ray diffraction (XRD) patterns were obtained using a diffractometer Siemens D5000, (KS Analytical Systems, Aubrey, TX, USA) operated at 35 kV and a current intensity of 20 mA. Samples were scanned in an angular range of 10–80° degrees (2θ). Thermogravimetric analyses (TGAs) were carried out on a TGA Q500 (TA Instruments, New Castle, PA, USA). Samples were heated from room temperature to 600 °C at 10 °C/min rate under nitrogen at 50 mL min^−1^. Pieces of compression cast plaques were cryo-fractured and coated with gold for scanning electron microscopy (Topocon South Asia Pte Ltd, Singapore, China) analysis. The SEM observations were realized directly on the external and cryo-fractured surfaces of the composites fabricated without ultrasound (W-U). SEM micrographs were obtained using a field emission scanning electron microscope (TopCon SM510) operated at 15 kV.

For evaluation of the combustion properties of composites, a dual cone calorimeter with a heat flux of 35 kW/m^2^ (Fire Testing Instrumentation, East Grinstead, UK) was used by following the method described by the ASTM E1354 standard. Sample dimensions (100 mm × 100 mm × 3 mm) were prepared by compression molding. The cone calorimeter was calibrated at 5 kW with methane flow, the flow in the extraction duct was 24 L/s, and the analyzer was calibrated with 20.95% oxygen. The heat flow used for assessing samples was 35 kW/m^2^. The sample was placed on an aluminum paper tray with the same sample dimensions and a 1 cm height, leaving the surface to evaluate a free area of 100 mm^2^. Then, the sample was placed in the sample holder, adjusting the distance between the cone and the surface of the sample to 25 mm, each sample was tested in duplicate. The flame retardancy index (FRI) is represented in Equation (1), and it is defined as the ratio of $THR\times \left(\frac{pHRR}{TTI}\right)\;$ between the neat polymer and the corresponding thermoplastic composite with flame-retardant additive [22].

(1) $$Flame\;Retardacy\;Index\;\left(FRI\right)=\frac{\lfloor THR\times \left(\frac{pHRR}{TTI}\right)\rfloor \;Neat\;Polymer}{\lfloor THR\times \left(\frac{pHRR}{TTI}\right)\rfloor Composite}\;$$

To determine the mechanical properties of the composites obtained in the extrusion process, the composites were injection molded in a Hi-Tech industrial injection machine (model HT-150) to obtain V-type samples. Tension tests were performed according to the ASTM D-638 and ASTM D-790 standards using a universal tensile machine (INSTRON model 4301) with a stretch rate of 5 mm/min^−1^.

## 3. Results and Discussion

### 3.1. X-ray Diffraction 

**Figure 2:**
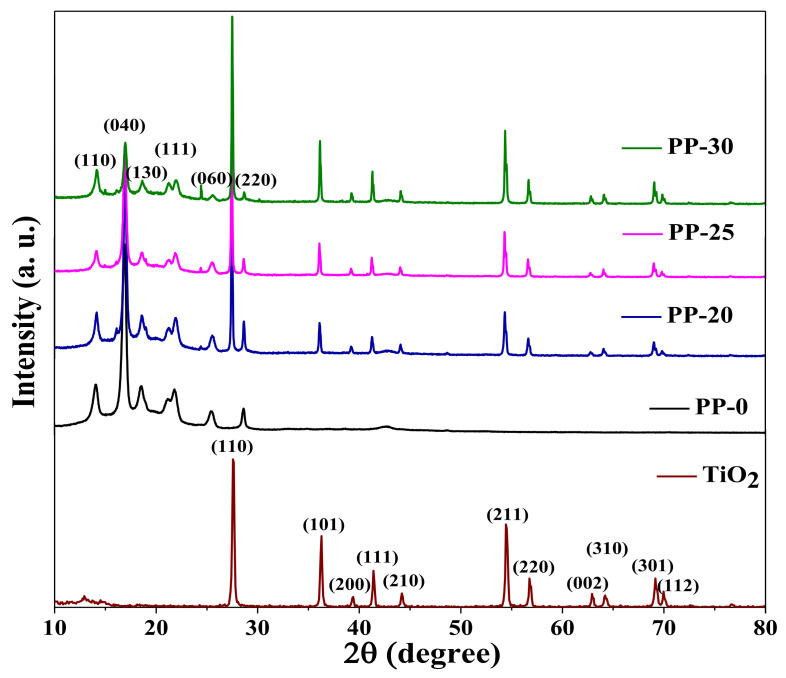
X-ray diffraction patterns of the composites PP–TiO_2_/lignin (PP-20, PP-25, and PP-30), polypropylene (PP-0), and TiO_2_.

Figure 2 shows the X-ray diffraction (XRD) patterns of the TiO_2_ and polypropylene (PP) nanoparticles with the aim of comparing the diffraction patterns of the base materials, with each of the composites obtained from PP–TiO_2_/lignin by the melt-extrusion process. The patterns identified in each of the composites obtained showed intense reflections at angles 14.23, 16.94, 18.58, 21.43, 25.66, and 28.65° corresponding to the planes (110), (040), (130), (111), (060), and (220) of the crystalline phase of the PP [8,23]. In addition, signals could be observed for 2θ values of 27.56, 36.28, 39.41, 41.46, 44.17, 54.53, 56.98, 62.96, 64.03, 69.09, and 69.98° corresponding to the Miller planes (110), (101), (200), (111), (210), (211), (220), (002), (310), (301), and (112) of the rutile crystalline phase (TiO_2_) (JCDPS 21-1276), which confirms its incorporation into the polymeric matrix of the PP. However, it was not possible to determine the lignin phase due to the amorphous nature of the material, which lacks an ordered structure [24]. Furthermore, no increase in the intensity or width of the PP signals was observed, which suggests that the addition of TiO_2_ had no effect on the crystalline structure of the polymeric matrix, as shown in the literature [23]. 

### 3.2. Fourier Transform Infrared FTIR (ATR)

**Figure 3:**
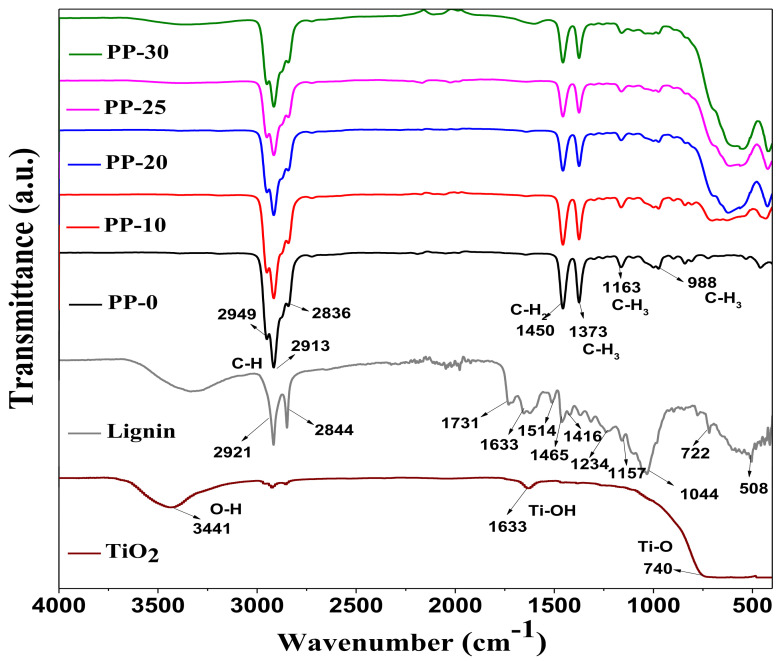
FTIR spectra of composites of PP–TiO_2_/lignin (PP-10, PP-20, PP-25, and PP-30), polypropylene (PP.0), lignin, and TiO_2_.

The results of the FTIR spectroscopy study are presented in Figure 3. For each of the composites (i.e., PP-10, PP-20, PP-25, and PP-30), the absorption signals typical of a polymer matrix of PP in the range of 2949–2836 cm^−1^ are shown. These signals or bands are associated with symmetrical and asymmetrical stretching of the C–H bonds present in the molecular structure of PP [8,25]. In addition, absorption bands at 1450, 1373, 1163, and 988 cm^−1^ corresponding to CH_2_ and CH_3_ were identified. The FTIR spectrum of TiO_2_ nanoparticles showed signals at 3441 cm^−1^ for the hydroxyl group (OH), 1633 cm^−1^ for the Ti–OH bond, and 740 cm^−1^ for the Ti–O bond [26,27]. For the lignin FTIR spectrum, adsorption signals at 2921 and 2844 cm^−1^ were localized that were related to the stretching of the C–H bonds of the aromatic methoxy groups (OCH_3_) and methyl and methylene groups present in the lignin molecule. A band located at 1731 cm^−1^ was associated with carbonyl groups present in the molecule; in addition, absorption bands at 1633, 1514, and 1465 cm^−1^ representing the vibrations of the aromatic skeleton of the molecule could also be observed; the localized band at 1416 cm^−1^ with less intensity was attributed to the deformation of the C–H bond combined with the vibrations of the aromatic ring of lignin. The signals localized below 1400 cm^−1^ were related to the C–O and C–H bonds present in the chemical structure of lignin [28,29]. The FTIR spectrum of each of the nanocomposites of PP/TiO_2_–lignin presented characteristic absorption signals of the polymer matrix (PP), TiO_2_ nanoparticles, and lignin; however, the latter presented very weak or almost null signals. Only in the composite PP-30 was it possible to observe a very weak absorption band located at 1604 cm^−1^ that could be associated to the interaction of the TiO_2_ nanoparticles and lignin. A similar study was reported by Guo et al., who observed an absorption band located at 1631 cm^−1^ which was associated to the interaction of hydrogen bonds between lignin surface groups and TiO_2_ nanoparticles [24]. In addition, in each FTIR spectrum of the composites, an absorption band centered at 560 cm^−1^ was located, which was attributed to the vibrations of Ti–O bonds that are characteristic of TiO_2_ nanoparticles. According to Poudel et al. [30], signals in the range of 700–420 cm^−1^ can be attributed to the vibrations of Ti–O bonds that represent the interaction of nanoparticles with the polymer matrix.

### 3.3. Thermogravimetric Analysis (TGA)

**Figure 4:**
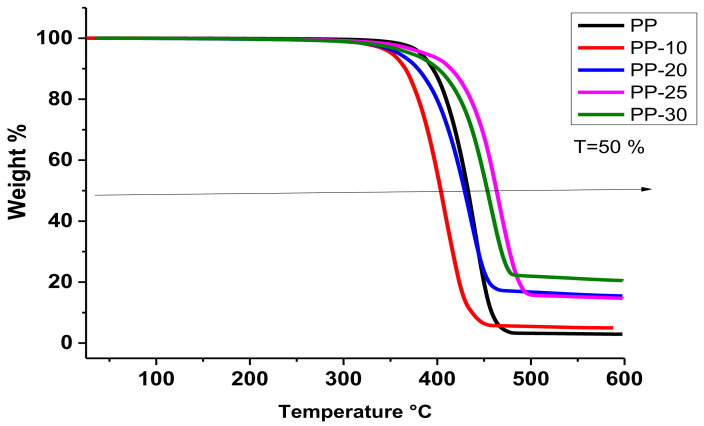
Thermograms of PP/TiO_2_/lignin composites obtained by extrusion.

In order to determine the thermal stability and weight loss with respect to temperature, thermogravimetric analyses was carried. Figure 4 shows the thermograms of the composites obtained and of the PP polymer, and it can be seen that the weight losses were in the temperature range between 309 and 489 °C.

For polymer PP, its weight loss started at 258 °C and ended at 482 °C, while the PP-10 sample showed a weight loss in a temperature range from 312 to 455 °C, and the PP-20 sample presented an important weight loss from 309 to 488 °C. The sample with a 25% load weight (i.e., PP-25) showed a weight loss temperature range from 335 to 488 °C, while the sample with the 30% load weight (i.e., PP-30) presented a weight loss temperature range of 323–489 °C. Samples PP-25 and PP-30 presented the highest thermal stability and contained the highest percentage by weight of lignin. Lignin has excellent thermal properties; its thermal decomposition temperature is reported at 450 °C [31]. The thermal behavior of the analyzed PP samples was consistent with that reported by Lukasz Klapiszewski et al. In 2016, they studied lignin–polypropylene composites and showed that low contents of lignin compounds are thermally less stable than pure polypropylene; kraft lignin supplied by Sigma–Aldrich was used in that study [15].

Table 2 shows the temperature at which 50% weight loss (T50) was achieved. The 10% and 20% loaded samples lost weight at lower temperatures (401 and 413 °C, respectively) in comparison to the unfilled PP polymer (431 °C). However, the PP-25 and PP-30 samples exhibited the highest thermal stability with T50 values of 448 and 453 °C, respectively. The residues at 500 °C of samples PP-10, PP-20, PP-25, and PP-30 according to the thermogravimetric study were 6%, 17%, 16%, and 21%, respectively (See Table 2). The values were similar to the percentage by weight of TiO_2_ used in each formulation, with the exception of the PP-20 sample, which was slightly larger. The PP-25 sample presented a value of 16 wt.% lower than that of the PP-20 sample; this was attributed to inhomogeneity of the samples.

PP and polyolefins (POs) do not contain polar groups in their structure; this feature limits their compatibility with polar polymers. Lignin has a strong polarity and functionality; thus, complete immiscibility with non-polar polyolefins would be expected. However, some studies suggest the opposite and claim that good dispersion and compatibility is achieved despite the different chemical structures of the two types of polymers, and the effect is more important when the amount of lignin added to the polymer is high. The thermal oxidative stability of PO can be improved considerably due to the fact that lignin phenolic OH groups are able to eliminate free radicals during the processing of polyolefins. Lignin can act as a stabilizer and protect the polymer matrix against oxidation [32,33,34,35].

In the PP-10 and PP-20 samples, the thermal stability effect decreased, probably due to the fact that the amount of lignin was not enough to prevent the thermal degradation of PP and that TiO_2_ did not act to stop thermal degradation. It has also been reported that when polypropylene/lignin compounds begin to degrade, carbonized aromatic radicals of lignin tend to reduce the rate of degradation of PP and produce char as a protective layer [36]. When there is a proper interface interaction, the particles are able to restrict the movement of the polymer chain, making it more difficult for polymer chains to break down at lower temperatures, leading to an increase in the material degradation temperature, and this has also been reported by Cabello-Alvarado et al. [8].

In the present study, the thermal stability of the PP matrix was only achieved in the PP-25 and PP-30 samples, where the charges (lignin and TiO_2_) were 25 and 30 wt.%; with these percentages, a good amount of phenolic OH groups is ensured and favor carbon structures that serve as protective layers during degradation.

### 3.4. Scanning Electron Microscopy (SEM)

**Figure 5:**
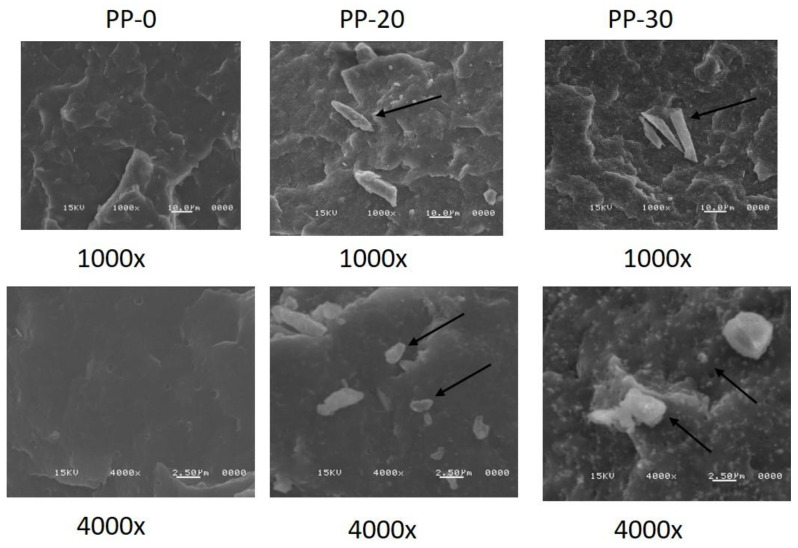
Scanning electron micrographs of PP-0, PP-20, and PP-30 at 1000× and 4000×.

Figure 5 shows SEM images of the PP polymer and the PP-20 and PP-30 composites. These composites were chosen because they had the highest thermal stability of the composites studied. Images with 1000× and 4000× resolutions were taken of analyzed samples. In these micrographs, it can be seen that by increasing the load content of TiO_2_ and lignin, there were small clusters in which the size of the agglomerated particles was much larger than the primary TiO_2_ particles (200 nm). In addition, other particles were homogeneously embedded in the PP matrix that had quasi-spherical shapes and sizes similar to the TiO_2_ NPs. 

A detailed analysis of the observed particle size suggests that the PP-20 sample had agglomerates that measured at their longest length from 1.6 to 23.4 microns, and quasi-spherical particles distributed in the PP matrix measured from 200 to 260 nm. For the PP-30 sample, the size of most agglomerates fluctuated between 1.9 and 6.2 microns, and only the micrograph presented in Figure 5 shows an agglomerate of 31.8 microns. The quasi-spherical particles had a size between 250 and 350 nm, and the size was slightly higher than those observed in PP-20. This microscopic evidence suggests that the interaction of lignin and TiO_2_ avoids the strong agglomeration of each filler into the PP matrix, favoring its dispersion during the melt-mixing extrusion process. It has been reported that PP nanocomposites with TiO_2_ present strong agglomerations from 4.0 wt.%, forming agglomerates of approximately 45 microns [37]. Composites of PP and modified lignin also present agglomeration from the concentration 5.0 wt.% [38]. PP composites obtained with a combination of TiO_2_ and lignin by the melt-mixed extrusion method led to nanocomposites with quasi-spherical particles well-dispersed in PP matrix to provide a larger interfacial area between the TiO_2_ nanoparticles and the surrounding polymer phase. PP/TiO_2_ nanocomposites with TiO_2_ contents greater than 2 wt.% suffer a strong agglomeration, and an increase in the average particle size of original TiO_2_ has been reported [8,39,40]; this phenomenon also occurs in nylon 6 nanocomposites with copper nanoparticles; in this polymer, the nanoparticles change their shape and size. The formation of aggregates of a micrometric size seems to be unavoidable and could be studied in more detail in the future. Zohrevand et al. proposed that micrometric aggregates can be dispersed during the melt extrusion process [41].

### 3.5. Cone Calorimetry

**Figure 6:**
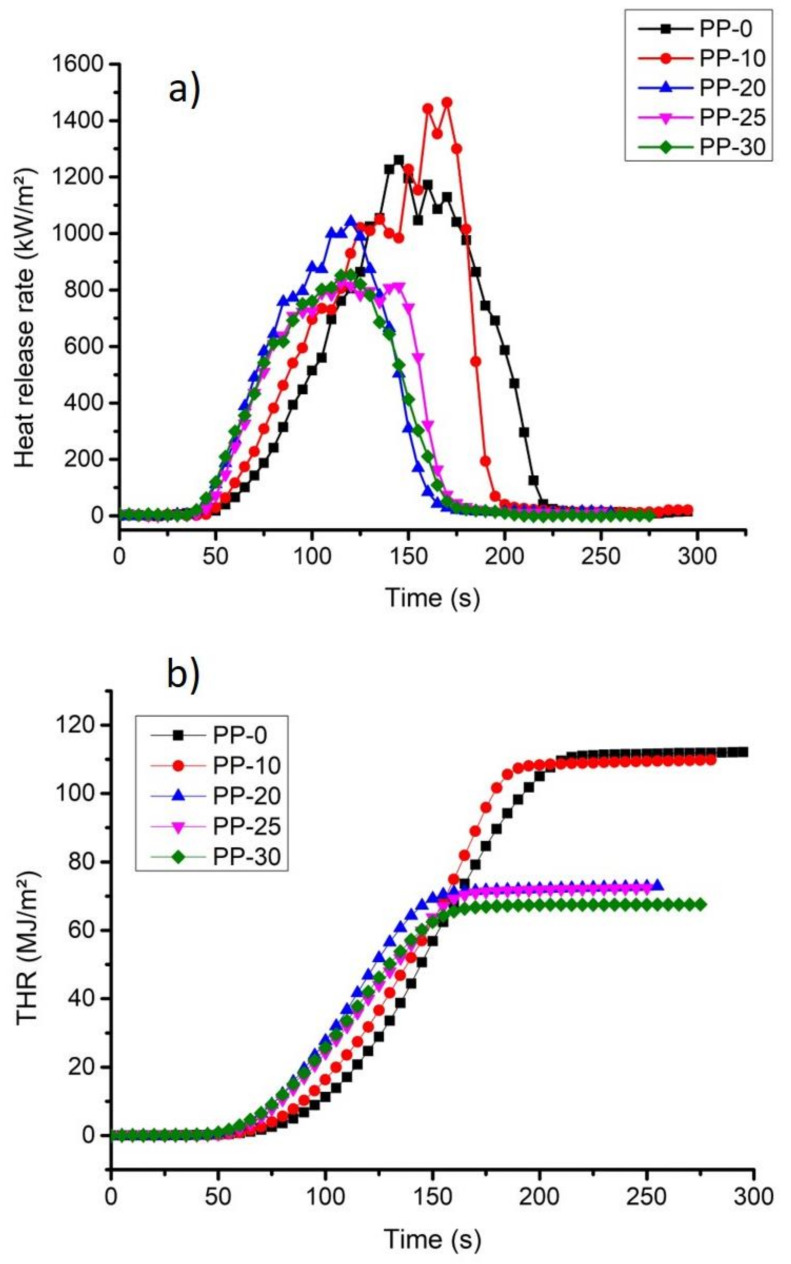
Calorimetric measurements: (**a**) comparison of the peaks of the heat release rate (HRR) and (**b**) THR curves for the results.

Figure 6a shows the HRR (heat release rate) results for PP polymer (1260 kW/m^2^) and the composites obtained at different load concentrations. In this graph, it can be seen that there is a decrease in the HRR peak for materials loaded with the TiO_2_/lignin additive. In particular, the PP-25 sample showed the best flame-retardant effect (827 kW/m^2^), decreasing the peak of HRR by 34.37% in relation to the pure polymer (Table 3). The fact that the peak was less intense implies lower flame propagation and less released heat. There are reports of polymer–lignin composite materials forming a carbonaceous layer that can prevent heat transfer and diffusion of small combustible products created by polymer degradation, also this layer reduces the rate of heat release, slowing down the process combustion [42].

THR (total heat release) curves for the PP polymer and the samples obtained are presented in Figure 6b. A THR value of 112 MJ/m^2^ was obtained for PP, while for composite materials, the THR decreased with increased loading (i.e., 109, 72.8, 72.3, and 67.5 MJ/m^2^), and the comparison of these values can be seen in Table 3. This trend is due to the thermal stability conferred by the TiO_2_/lignin charge on the polymer. It is known that TiO_2_ has a high decomposition temperature (700–800 °C) and an oxygen index of 29, and it has also been widely used as an anti-flame additive alone or in combination with other additives [43]. Bamboo substrates were covered with TiO_2_ and ZnO nanoparticles, significantly improving their flame-retardant properties, which is due to the formation of a network of TiO_2_ particles derived from the nanometric size of the particles, thus increasing their anti-flame property [44].

**Figure 7:**
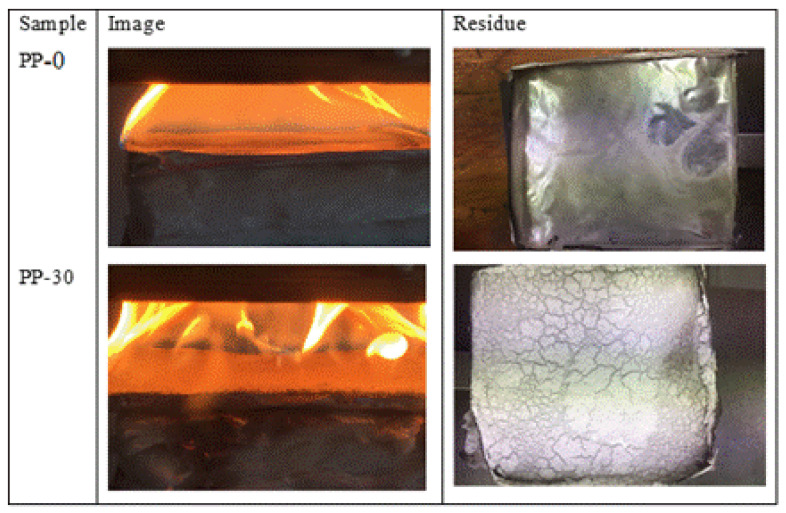
Digital photos of PP-0 and PP-30 after cone calorimeter measurements.

Figure 7 shows images of the calorimetric cone and the residue that was obtained after evaluating the PP-0 and PP-30 samples. The pure PP did not leave any residue on the aluminum tray, while the sample tray PP-30 showed residue that adhered to its entire surface.

In order to quantify the flame-retardancy performance of the different formulations studied, the FRI was calculated [10,22]. The FRI values presented in Table 4 show that the new composites can be classified as good based on the proposed criteria [22].

### 3.6. Mechanical Properties

The tensile and flexural properties of the PP-0, PP-10, PP-20, PP-25, and PP-30 composites are shown in Table 5. It can be seen that the tensile strength and elongation at break were diminished with addition lignin and TiO_2_; the results are those expected if we consider that TiO_2_ and lignin fillers tend to form large agglomerates in PP composites and are in accordance with some reported studies [45,46]; but other authors report an increase in properties, the difference in the results is attributed to the synthesis process. For example, Abdelwahab et al. reported that the tensile strength of the composite containing 20 wt.% lignin increased from 21.4 to 24.6 MPa, while the composite with 30 wt.% of lignin contents increased from 23.1 to 26.5 MPa; other properties, such as flexural modulus and impact strength, did not show significant changes. They used three different compatibilizers for better adhesion to the polymer matrix, but not in all cases was this property improved [47]. In the present study, the synthesis of composites of PP with two fillers and a high agglomeration capacity was carried out in a one-step process, avoiding the use of compatibilizers aiming to maintain an economic and scalable process at an industrial level

On the other hand, it can be seen in Table 5 that the flexural modulus of composites increased gradually with increasing lignin and TiO_2_ content and the flexural strength decreases slightly. It was clear that the presence of agglomerates detected on the composites’ surface by SEM affected the mechanical properties, but it should be noted that the composites also presented TiO_2_ particles with particle sizes very similar to primary TiO_2_ samples. This suggests that lignin interacts with TiO_2_, reducing its strong agglomeration capacity, as has been reported [37]. In addition, it was possible to detect by SEM surfaces free of holes or cracks, which indicates a good dispersion of the fillers. The results obtained suggest that it is possible to synthesize well-dispersed PP/TiO_2_/lignin composites with improved mechanical properties, reducing the amount of TiO_2_ and lignin in the formulations, as some reports suggest [37], another possible solution is to modify the process conditions with the purpose of breaking the agglomerates [41].

## 4. Conclusions

This paper presented the synthesis of composites based on PP/TiO_2_/lignin. The results obtained suggest that lignin can be considered an excellent alternative to be used in environmentally friendly fire safety plastics. All composites were synthesized in one-step by melt-mixing extrusion. The presence of TiO_2_ in the PP polymer matrix was detected in XRD; but lignin has an amorphous nature and did not show any peak in this study. The analyses of the PP-30 sample using FTIR showed evidence of all components (i.e., lignin, TiO_2_, and PP). SEM analysis showed uniform dispersion of TiO_2_ particles and lignin fibers, and the synthesis method used led to composites with few agglomerates. Because of this, the composites presented good results as flame retardants; specifically, the PP-25 and PP-30 composites both presented a reduction in HRR and THR, probably due to the synergy of lignin and TiO_2_, which managed to form a carbon layer that did not allow the diffusion of oxygen and heat, creating a barrier effect for combustion. It is important to highlight that the PP-30 sample presented an FRI value of 1.93 and represents one of the highest values reported for PP composites. In addition, the composites presented good thermal stability, which helps its possible application as green flame retardant.

## Figures and Tables

**Table 1:** Identification of the PP/TiO_2_/lignin composites with different percentages of additives (10, 20, 25, and 30 wt.%).

Sample Identification	PP Content (g)	Weight of Additive (g) TiO_2_	Weight of Additive (g) Lignin	Weight Percent of Additive (%)	Total Weight (g)
PP-0	300	0	0	0	300
PP-10	270	20	10	10	300
PP-20	240	40	20	20	300
PP-25	225	50	25	25	300
PP-30	210	60	30	30	300

**Table 2:** Temperature at 50% weight loss and residue at 550 °C of the PP/TiO_2_/lignin composites.

Sample	Temperature at 50% Weight Loss, T_50%_ °C	Residue at 550 °C (%)
PP-0	429	0
PP-10	401	6
PP-20	413	17
PP-25	448	16
PP-30	453	21

**Table 3:** Data of the cone calorimetry test of the samples analyzed.

Sample	Peak HRR (kW/m^2^)	THR (MJ/m^2^)	Reduction % HRR	Reduction % THR
PP-0	1260 ± 0.40	11.02 ± 0.72	-	-
PP-10	1463 ± 0.67	109.0 ± 0.40	16.11 ± 0.42	2.68 ± 0.4
PP-20	1041 ± 0.90	72.9 ± 0.50	17.39 ± 0.26	35.72 ± 0.2
PP-25	827 ± 0.34	72.3 ± 0.30	34.37 ± 0.37	35.45 ± 0.2
PP-30	853 ± 0.25	67.5 ± 0.12	32.31 ± 0.10	39.74 ± 0.6

**Table 4:** Flame retardancy index of PP/TiO_2_/lignin composites.

Sample	Fire Retardancy Index
PP-10	0.87
PP-20	1.45
PP-25	1.76
PP-30	1.93

**Table 5:** Tensile and flexural properties of the composites.

Sample	Tensile Strength (MPa)	Elongation at Break (%)	Flexural Strength (MPa)	Flexural Modulus (GPa)
PP-0	24.7 ± 0.25	218 ± 1.2	42.2 ± 1.79	1.26 ± 4.7
PP-10	20.8 ± 1.79	10.8 ± 0.2	39.4 ± 0.61	1.33 ± 1.6
PP-20	18.2 ± 2.21	4.9 ± 2.5	36.4 ± 0.91	1.34 ± 2.5
PP-25	13.7 ± 1.58	4.8 ± 4.12	33 ± 1.28	1.35 ± 3.0
PP-30	12.7 ± 1.93	3.1 ± 2.36	39.6 ± 1.51	1.52 ± 4.8

## Data Availability

Not applicable.

## References

[1] https://conapci.org/incendios-urbanos-en-mexico/.

[2] Rejeesh C.R., Saju K.K. (2017). Methods and materials for reducing flammability behaviour of coir fibre based composite boards: A review. Mater. Today Proc..

[3] Kemmlein S., Herzke D., Law R.J. (2009). Brominated flame retardants in the European chemicals policy of REACH—Regulation and determination in materials. J. Chromatogr. A.

[4] Jadhav S.D.A. (2018). Review of non-halogenated flame retardant. Pharma Innov..

[5] Cabello-Alvarado C., Andrade-Guel M., Ávila-Orta C.A., Gamero-Melo P., Reyes-Rodríguez P.Y., Quiñones-Jurado Z.V., Cadenas-Pliego G., Bartolo-Pérez P., Soriano-Corral F., Covarrubias-Gordillo C. (2021). Composites based on nylon 6/clinoptilolite by ultrasound-assisted extrusion for enhanced flame retardant and mechanical properties. Polym. Bull..

[6] Khezami L., Chetouani A., Taouk B., Capart R. (2005). Production and characterisation of activated carbon from wood components in powder: Cellulose, lignin, xylan. Powder Technol..

[7] He W., Song P., Yu B., Fang Z., Wang H. (2020). Flame retardant polymeric nanocomposites through the combination of nanomaterials and conventional flame retardants. Prog. Mater. Sci..

[8] Cabello-Alvarado C., Reyes-Rodríguez P., Andrade-Guel M., Cadenas-Pliego G., Alvarez M.P., Cruz-Delgado V., Melo-López L., Quiñones-Jurado Z., Ávila-Orta C. (2019). Melt-mixed thermoplastic nanocomposite containing carbon nanotubes and titanium dioxide for flame retardancy applications. Polymers.

[9] Wu W., Zhao W., Gong X., Sun Q., Cao X., Su Y., Yu B., Li R.K., Vellaisamy R.A. (2022). Surface decoration of Halloysite nanotubes with POSS for fire-safe thermoplastic polyurethane nanocomposites. J. Mater. Sci. Technol..

[10] Liu C., Yang D., Sun M., Deng G., Jing B., Wang K., Shi Y., Fu L., Feng Y., Lv Y. (2022). Phosphorous-Nitrogen flame retardants engineering MXene towards highly fire safe thermoplastic polyurethane. Compos. Comun..

[11] Wu W., He H., Liu T., Wei R., Cao X., Sun Q., Venkatesh S., Yuen K.K.R., Roy V.A., Li R.K.Y. (2018). Synergetic enhancement on flame retardancy by melamine phosphate modified lignin in rice husk ash filled P34HB biocomposites. Compos. Sci. Technol..

[12] Ji M., Li J., Li F., Wang X., Man J., Li J., Zhang C., Peng S. (2022). A biodegradable chitosan-based composite film reinforced by ramie fibre and lignin for food packaging. Carbohydr. Polym..

[13] Borysiak S. (2013). Fundamental studies on lignocellulose/polypropylene composites: Effects of wood treatment on the transcrystalline morphology and mechanical properties. J. Appl. Polym. Sci..

[14] Borysiak S. (2015). The thermo-oxidative stability and flammability of wood/polypropylene composites. J. Therm. Anal. Calorim..

[15] Klapiszewski Ł., Bula K., Sobczak M., Jesionowski T. (2016). Influence of processing conditions on the thermal stability and mechanical properties of PP/silica-lignin composites. Int. J. Polym. Sci..

[16] Pongsa U., Jamesang O., Sangrayub P., Lumsakul P., Kaweegitbundit P., Mookam N. (2021). Flammability of Short Agro-Waste Pineapple Leaf Fiber Reinforced Polypropylene Composite Modified with Diammonium Phosphate Flame Retardant and Titanium Dioxide. Fibers Polym..

[17] Wu K., Xu S., Tian X.Y., Zeng H.Y., Hu J., Guo Y.H., Jian J. (2021). Renewable lignin-based surfactant modified layered double hydroxide and its application in polypropylene as flame retardant and smoke suppression. Int. J. Biol. Macromol..

[18] Yu Y., Fu S., Song P., Luo X., Jin Y., Lu F., Wu Q., Ye J. (2012). Functionalized lignin by grafting phosphorus-nitrogen improves the thermal stability and flame retardancy of polypropylene. Polym. Degrad. Stab..

[19] Yang H., Yu B., Xu X., Bourbigot S., Wang H., Song P. (2020). Lignin-derived bio-based flame retardants toward high-performance sustainable polymeric materials. Green Chem..

[20] El-dessouky H.M., Lawrence C.A. (2011). Nanoparticles dispersion in processing functionalized PP/TiO_2_ nanocomposite: Distribution and properties. J. Nanopart. Res..

[21] Cassagnau P., Legare V., Fenouillot F. (2007). Reactive processing of thermoplastic polymer: A review of the fundamental aspect. Int. Polym. Process..

[22] Sallem-Idrissi N., Sclavons M., Debecker D.P., Devaux J. (2016). Miscible raw lignin/nylon 6 blends: Thermal and mechanical performances. J. Appl. Polym. Sci..

[23] Wang S., Ajji A., Guo S., Xiong C. (2017). Preparation of microporous polypropylene/titanium dioxide composite membranes with enhanced electrolyte uptake capability via melt extruding and stretching. Polymers.

[24] Guo D., Zhang J., Sha L., Liu B., Zhang X., Zhang X., Xue G. (2020). Preparation and characterization of lignin-TiO_2_ UV-shielding composite material by induced synthesis with nanofibrillated cellulose. Bioresources.

[25] Liu L., Qian M., Song P., Huang G., Yu Y., Fu S. (2016). Fabrication of Green ligning-based flame retardants for enhancing the thermal and fire retardancy properties of polypropylene/Wood composites. Sustain. Chem. Eng..

[26] Liu Z., Jian Z., Fang J., Xu X., Zhu X., Wu S. (2012). Low-temperature reverse microemulsion synthesis, characterization, and photocatalytic performance of nanocrystalline titanium dioxide. Int. J. Photoenergy..

[27] Leon A., Reuquen P., Garin C., Segura R., Vargas P., Zapata P., Orihuela P. (2017). FTIR and Raman characterization of TiO_2_ nanoparticles coated with polyethylene glycol as carrier for 2-methoxyestradiol. Appl. Sci..

[28] Boeriu C., Bravo D., Gosselink R.J., van Dam J.E. (2004). Characterization of structure-dependent functional properties of lignin with infrared spectroscopy. Ind. Crops Prod..

[29] Nandanwar R.A., Chaudhari A.R., Ekhe J.D. (2016). Nitrobenzene oxidation for isolation of value added products from industrial waste lignin. J. Chem. Biol. Phys. Sci..

[30] Poudel B.R., Aryal R.L., Bhattarai S., Koirala A.R., Gautam S.J., Ghimire K.N.A., Pant B., Park M., Paudyal H., Pokhrel M.R. (2020). Agro-waste derived biomass impregnated with TiO_2_ as a potential adsorbent for removal of as (III) from water. Catalysts.

[31] Chávez-Sifontes M., Domine M.E. (2013). Lignina, estructura y aplicaciones: Métodos de despolimerización para la obtención de derivados aromáticos de interés industrial. Av. En Cienc. E Ing..

[32] Kun D., Pukánszky B. (2017). Polymer/lignin blends: Interactions, properties, applications. Eur. Polym. J..

[33] Levon K., Huhtala J., Malm B., Lindberg J.J. (1987). Improvement of the thermal stabilization of polyethylene with lignosulphonate. Polymer.

[34] Gregorova A., Košíková B., Staško A. (2007). Radical scavenging capacity of lignin and its effect on processing stabilization of virgin and recycled polypropylene. J. Appl. Polym. Sci..

[35] Jeong H., Park J., Kim S., Lee J., Cho J.W. (2012). Use of acetylated softwood kraft lignin as filler in synthetic polymers. Fibers Polym..

[36] Li J., Li B., Zhang X., Su R. (2001). The study of flame retardants on thermal degradation and charring process of manchurian ash lignin in the condensed phase. Polym. Degrad. Stab..

[37] Aydemir D., Uzun G., Gumuş H., Yildiz S., Gumuş S., Bardak T., Gunduz G. (2016). Nanocomposites of polypropylene/nano titanium dioxide: Effect of loading rates of nano titanium dioxide. Mater. Sci..

[38] Maldhure A.V., Chaudhari A.R., Ekhe J.D. (2011). Thermal and structural studies of polypropylene blended with esterified industrial waste lignin. J. Therm. Anal. Calorim..

[39] Orellana F., Lisperguer J., Nuñez C. (2014). Synthesis and characterization of polypropylene-silica, alumina and titania nanoparticles, prepared by melting. J. Chil. Chem. Soc..

[40] Sierra-Ávila R., Pérez-Alvarez M., Valdez-Garza J., Avila-Orta C.A., Jiménez-Regalado E.J., Mata-Padilla J.M., Soto-Castruita E., Cadenas-Pliego G. (2018). Synthesis and Thermomechanical Characterization of Nylon 6/Cu Nanocomposites Produced by an Ultrasound-Assisted Extrusion Method. Adv. Mater. Sci. Eng..

[41] Zohrevand A., Ajji A., Mighri F. (2014). Morphology and properties of highly filled iPP/TiO_2_ nanocomposites. Polym. Eng. Sci..

[42] Bourbigot S., Lebras M.L., Delobel R., Breant P., Tremillon J.M. (1995). Carbonization mechanisms resulting from intumescence-part II. Association with an ethylene terpolymer and the ammonium polyphosphate pentaerythritol fire-retardant system. Carbon.

[43] Lam Y.L., Kan C.W., Yuen C.W.M. (2011). Effect of titanium dioxide on the flame-retardant finishing of cotton fabric. J. Appl. Polym. Sci..

[44] Ren D., Li J., Xu J., Wu Z., Bao Y., Li N., Chen Y. (2018). Efficient Antifungal and Flame-Retardant Properties of ZnO-TiO_2_-Layered Double-Nanostructures Coated on Bamboo Substrate. Coatings.

[45] Kamrannejad M.M., Hasanzadeh A., Nosoudi N., Mai L., Babaluo A.A. (2014). Photocatalytic degradation of polypropylene/TiO_2_ nano-composites. Mater. Res..

[46] Chen F., Dai H., Dong X., Yang J., Zhong M. (2011). Physical properties of lignin-based polypropylene blends. Polym. Compos..

[47] Abdelwahab M.A., Misra M., Mohanty A.K. (2019). Injection molded biocomposites from polypropylene and lignin: Effect of compatibilizers on interfacial adhesion and performance. Ind. Crops Prod..

